# Supplementary material for: Triple combination therapy of favipiravir plus two monoclonal antibodies eradicates influenza virus from nude mice
Source: Commun Biol. 2020 May 7;3:219. doi: 10.1038/s42003-020-0952-y (PMC7205604; doi:10.1038/s42003-020-0952-y)
Supplement: Supplementary file 1 — Reporting summary [file 42003_2020_952_MOESM1_ESM.pdf]

nature research

Corresponding author(s): Yoshihiro Kawaoka and Seiya Yamayoshi

Last updated by author(s): Dec 24, 2019

Reporting Summary

Nature Research wishes to improve the reproducibility of the work that we publish. This form provides structure for consistency and transparency in reporting. For further information on Nature Research policies, see Authors & Referees and the Editorial Policy Checklist .

Statistics

For all statistical analyses, confirm that the following items are present in the figure legend, table legend, main text, or Methods section.

n/a	Confirmed	
☐	☒	The exact sample size (*n*) for each experimental group/condition, given as a discrete number and unit of measurement
☐	☒	A statement on whether measurements were taken from distinct samples or whether the same sample was measured repeatedly
☐	☒	The statistical test(s) used AND whether they are one- or two-sided<br>*Only common tests should be described solely by name; describe more complex techniques in the Methods section.*
☐	☒	A description of all covariates tested
☐	☒	A description of any assumptions or corrections, such as tests of normality and adjustment for multiple comparisons
☐	☒	A full description of the statistical parameters including central tendency (e.g. means) or other basic estimates (e.g. regression coefficient) AND variation (e.g. standard deviation) or associated estimates of uncertainty (e.g. confidence intervals)
☐	☒	For null hypothesis testing, the test statistic (e.g. *F*, *t*, *r*) with confidence intervals, effect sizes, degrees of freedom and *P* value noted<br>*Give P values as exact values whenever suitable.*
☒	☐	For Bayesian analysis, information on the choice of priors and Markov chain Monte Carlo settings
☒	☐	For hierarchical and complex designs, identification of the appropriate level for tests and full reporting of outcomes
☒	☐	Estimates of effect sizes (e.g. Cohen's *d*, Pearson's *r*), indicating how they were calculated

Our web collection on statistics for biologists contains articles on many of the points above.

Software and code

Policy information about availability of computer code

Data collection	Sequence data were collected using ABI Prism 3130xl Genetic Analyzer data collection software (version 3.0).
Data analysis	GENETYX genetic information processing software (version 13) was used for sequence alignment. ATGC sequence assembly software (version 7.0) was used for sequence assemblies. Graphpad Prism (version 5) was used for calculating IC50 values and statistical analysis. Structural analysis was performed by using the PyMOL molecular graphics system.

For manuscripts utilizing custom algorithms or software that are central to the research but not yet described in published literature, software must be made available to editors/reviewers. We strongly encourage code deposition in a community repository (e.g. GitHub). See the Nature Research guidelines for submitting code & software for further information.

Data

Policy information about availability of data

All manuscripts must include a data availability statement. This statement should provide the following information, where applicable:

- Accession codes, unique identifiers, or web links for publicly available datasets
- A list of figures that have associated raw data
- A description of any restrictions on data availability

All data analyzed during this study are included in this article. The datasets generated and analyzed during the current study are available from the corresponding author on reasonable request.

Field-specific reporting

Please select the one below that is the best fit for your research. If you are not sure, read the appropriate sections before making your selection.

Life sciences study design

All studies must disclose on these points even when the disclosure is negative.

Sample size	No sample-size calculataion was performed.
Data exclusions	No data were excluded.
Replication	In vitro assays were performed in 2 independent experiments. The in vivo protection test was performed with four mice per experimental group. All available data are included in the manuscript.
Randomization	No method of randomization was used to determine how the animals were allocated to the experimental groups and processed in this study.
Blinding	No blinding was performed.

Reporting for specific materials, systems and methods

We require information from authors about some types of materials, experimental systems and methods used in many studies. Here, indicate whether each material, system or method listed is relevant to your study. If you are not sure if a list item applies to your research, read the appropriate section before selecting a response.

Materials & experimental systems

n/a	Involved in the study
☐	☒ Antibodies
☐	☒ Eukaryotic cell lines
☒	☐ Palaeontology
☐	☒ Animals and other organisms
☒	☐ Human research participants
☒	☐ Clinical data

Methods

n/a	Involved in the study
☒	☐ ChIP-seq
☒	☐ Flow cytometry
☒	☐ MRI-based neuroimaging

Antibodies

Antibodies used	All human antibodies used in this study were made recombinantly by cloning antibody heavy and light chains into mammalian expression vectors. Antibodies were produced in mammalian cells (Expi293 cells) by transient transfection of expression vectors and purified by affinity chromatography.
Validation	Sequence, specificity, and function of the human antibodies were published (Dreyfus, C. et al. 2012 Science; Yasuhara et al. 2018 Front Microbiol)

Eukaryotic cell lines

Policy information about cell lines

Cell line source(s)	Madin-Darby canine kidney (MDCK) cells are available in our lab; Expi293F cells (Thermo Fisher Scientific)
Authentication	The DNA fingerprinting method was used to show that the MDCK cells have the same origin as the MDCK (CCL-34) obtained from ATCC.
Mycoplasma contamination	All cell lines were regularly tested for mycoplasma contamination by using PCR and were confirmed to be mycoplasma-free.
Commonly misidentified lines (See ICLAC register)	No commonly misidentified cell lines were used.

Animals and other organisms

Policy information about studies involving animals; ARRIVE guidelines recommended for reporting animal research

Laboratory animals	Five-week-old female BALB/c-nu/nu mice (Japan SLC) were used in the study.
Wild animals	n.a.
Field-collected samples	n.a.

Ethics oversight

All experiments with mice were performed in accordance with the University of Tokyo's Regulations for Animal Care and Use and were approved by the Animal Experiment Committee of the Institute of Medical Science, the University of Tokyo.

Note that full information on the approval of the study protocol must also be provided in the manuscript.
